# Functional Recovery in Chronic Stage of Spinal Cord Injury by Neurorestorative Approach: A Case Report

**DOI:** 10.1155/2014/404207

**Published:** 2014-03-11

**Authors:** Alok Sharma, Hemangi Sane, Dipti Khopkar, Nandini Gokulchandran, V. C. Jacob, Joji Joseph, Prerna Badhe

**Affiliations:** ^1^Department of Medical Services and Clinical Research, NeuroGen Brain and Spine Institute, Surana Sethia Hospital and Research Centre, Suman Nagar, Sion Trombay Road, Chembur, Mumbai 400071, India; ^2^Department of Research and Development, NeuroGen Brain and Spine Institute, Surana Sethia Hospital and Research Centre, Suman Nagar, Sion Trombay Road, Chembur, Mumbai 400071, India; ^3^Department of Neurorehabilitation, NeuroGen Brain and Spine Institute, Surana Sethia Hospital and Research Centre, Suman Nagar, Sion Trombay Road, Chembur, Mumbai 400071, India

## Abstract

Spinal cord injury (SCI) at an early age can be debilitating for the child's growth. Current treatments show a level of stagnancy, after which the recovery is minimal. Cellular therapy is an emerging area of research and has been found to possess many benefits in the previous studies. Transplantation of autologous bone marrow mononuclear cells (BMMNCs) has demonstrated therapeutic potential for many neurological conditions, including spinal cord injury. Here we report a case of 6-year-old girl with traumatic SCI at the level of C7-D1 4 years back, who underwent 2 doses of cell transplantation with autologous BMMNCs with an interval of 6 months along with standard rehabilitation. The patient did not have any major or minor side effects. The patient showed clinical improvements throughout the 6 months after transplantation, which was assessed using Functional Independence Measure (before: 82, after: 101 out of 126). There were patchy areas of sensory gain in bilateral feet recorded, with improvements in the bladder sensation and control. Improved gait was seen as a result of better strength in abdominals and back extensors. The fact that there was functional improvement in the chronic plateau phase indicates the potential of cell therapy in chronic SCI. Further clinical studies are warranted.

## 1. Introduction

Spinal cord injury (SCI) at an early age is very debilitating for the child in every aspect of life, since it hinders their physical, emotional, and social growth [1]. The major cause of this event is road traffic accidents. Other causes include falls, sports injuries, or violence [2]. Spinal cord injuries are complex, as the central nervous system has a limited capability of regeneration [3]. In spinal cord injury, there is a partial or total disruption of the ascending and descending tracts, which carry information to and from the brain. This poses a great challenge for the health professionals involved in the rehabilitation team, as regeneration needs to happen in between the nerve fibers of the two stumps of the cord [2]. The levels of spinal cord injury may vary, with cervical spine injury leading to quadriplegia; and thoracic and lumbar injuries leads to paraplegia [2]. One of the recent and developing options in improving the condition of patients with neurologically compromised conditions, including spinal cord injury, is cellular therapy. Autologous bone marrow cells have been deemed to be relatively safe option for treating this group of patients. Their effects have been seen in many of the ongoing clinical studies. They are free from any ethical issues, as they are derived from the same patients bone marrow, unlike the embryonic and umbilical cord cells. Autologous cell therapy is also free from any immunologic reactions [4]. The physiology of how cell therapy works has been shown in the previous animal studies done. The cells tend to migrate from the site of injection to the site of injury. Their effects are seen as a result of a few physiological processes like enhanced angiogenesis, which contributes to neovascularisation at the site of injury, by producing signaling molecules such as fibroblast growth factor (FGF2) and vascular endothelial growth factor (VEGF). They also help in remodeling, prevent apoptosis, and decrease inflammation at the site of injury [5].

Based on the previous research and the rationale, following is a case report of a 6-year-old girl with traumatic spinal cord injury treated with autologous bone marrow mononuclear cells (BMMNCs).

## 2. Case Presentation

### 2.1. A Case Report

**Figure 1:**
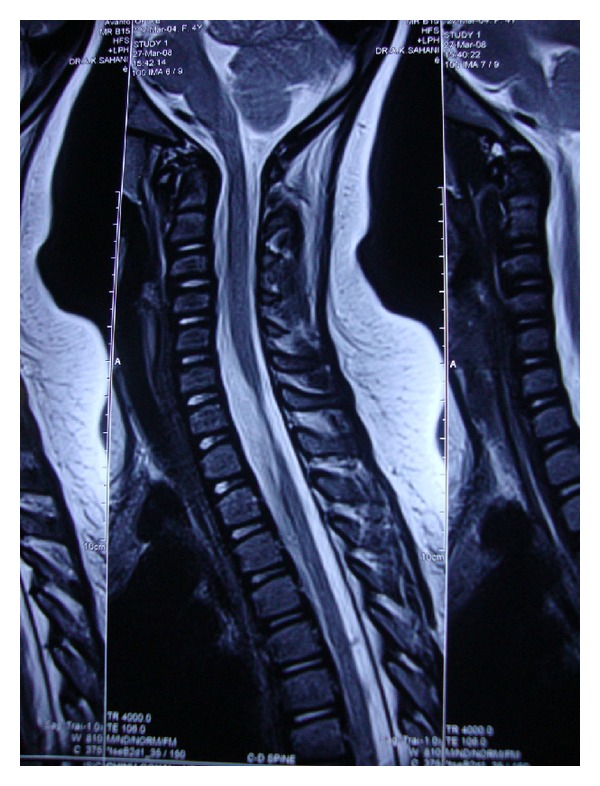
MRI of spine showing injury from C7 to D1.

A 6-year-old girl presented with a history of road traffic accident 4 years ago, causing trauma to the spinal cord. Soon after the trauma, paralysis of both lower limbs and hands was noticed by her parents. The child underwent extensive rehabilitation leading to complete recovery of her upper limbs, but developed a plateau in her recovery phase below the level of injury. On detailed assessment prior to the cell therapy, she exhibited neurological features like hypotonia and hyporeflexia in bilateral lower limbs. Strength of grade 5 in bilateral upper limbs and grade 0 in bilateral lower limbs was recorded. Total sensory loss below D10 level was present. Urinary incontinence was reported with poor urine control. Magnetic resonance imaging (MRI) showed focal myelomalacia from C7 to D1 in the form of focal atrophy of the cord extending from C7 to D1 with altered signals at these levels; see Figure 1. Functionally, she was partially dependent on her mother for activities of daily living (ADL), mainly mobility. She was able to stand and walk with a walker and Hip Knee Ankle Foot Orthosis (HKAFO) with difficulty and many compensatory strategies. Trunk control was poor in standing. The patient underwent two doses of cell therapy with a gap of 6 months between the two. She scored 82 out of 126 on Functional Independence Measure (FIM). On American Spinal Injury Association (ASIA) scale, she was at level A.

### 2.2. Method

#### 2.2.1. 1st Dose

Based on inclusion criterion as per the World Medical Associations Helsinki Declaration, the patient was selected for intervention. The treatment protocol is approved by the Institutional Committee for Stem Cell Research and Therapy (IC-SCRT). Prior to admission, a signed informed consent was obtained from the parents. Granulocyte-Colony Stimulating Factor (G-CSF) (300 mcg) injections were administered subcutaneously, 48 hours and 24 hours prior to bone marrow aspiration. Autologous bone marrow mononuclear cells (MNCs) transplantation was done for the child. With the patient in supine position, local anesthesia was given in the region of the right anterior superior iliac spine with sedation. Following this, using a bone marrow aspiration needle, 100 mL of bone marrow was aspirated and collected in heparinized tubes and transported to the laboratory. In the laboratory, the MNCs were separated by the density gradient method. The cells were sent for CD34 counts. Total cell count was 83 × 10^6^, out of which 96% cells were viable. CD34 count was 1.23%. The cells were transported back to the OT in a sterile cool container. The patient was positioned in lateral position and, using a lumbar puncture needle and cerebrospinal fluid (CSF) drainage set, the thecal sac was punctured in the L4-L5 space. A catheter was introduced into the thecal sac and the cells were injected through the catheter. The catheter was then withdrawn after the cells were injected. Methylprednisolone 500 mg in 100 mL saline was given intravenously simultaneously during the injection.

After the cellular therapy, patient underwent rigorous physiotherapy and occupational therapy.

One week after cell therapy, a reassessment was conducted which showed some clinical improvements. Sensory improvement was seen in both of the lower limbs, mainly touch in both of the soles of her feet and patchy areas in the legs as compared to no sensations previous to the cell therapy. She also exhibited improvement in her urinary control, as she was able to hold urine for one and half hours as compared to poor control previously. With respect to ambulation, her gait was significantly improved using HKAFO. Improved strength of back extensors and abdominals was also seen. In view of improvements after cell therapy and neurorehabilitation, the patient underwent 2nd dose after a span of 6 months. During these 6 months, the mother continued her exercises at home.

#### 2.2.2. 2nd Dose

The patient underwent the same procedure of cell therapy.

After 2nd dose of cell therapy, patient underwent physiotherapy and occupational therapy for one week. After one week reassessment was done. Urinary control improved; thus she could hold for 2 to 2 and 1/2 hours and could pass voluntarily. There was a significant improvement in her gait, but with increased lordosis. She showed minimal control of bowel. Use of diapers was restricted only when outdoors, thus reducing her dependency on them. Her sitting and standing balance improved. She was now able to do tasks like bending down and picking up objects from floor in standing position, which was difficult for her earlier. ASIA grade remained unchanged at level A. She now scored 101 out of 126 on FIM as compared to 82 previously with training for stair climbing, which was significant.

The child showed increased duration in her urinary control from 1 (1/2) to 2 (1/2) hours, thus reducing her dependency on diapers. As a result of improved back extensors and abdominals, there was a significant improvement in her gait. Her static and dynamic balance improved. Antigravity positions and activities were easier for her, which was reflected in her FIM scores.

## 3. Discussion

Pediatric spinal cord injuries are rare and the management is not well established. SCI at such an early age can be very debilitating for the child. This approach was an attempt of cellular therapy to promote recovery and functional independence.

As the patient opted for cell therapy four years after the injury, the injured spinal cord atrophy was significant. The clinical changes seen as a result of cumulative effect of the 2 doses of cell therapy and rehabilitation may be attributed to various mechanisms of neurorestoration. The MNCs administrated intrathecally are automatically driven to site of injury. They home onto the damaged cord and transdifferentiate to form neural reconnections [6]. Also the paracrine effect helps suppress apoptosis and inflammation and facilitate release of VGEF that signals local repair. The angiogenesis and neovascularisation brought about by the MNCs at the site of injury of spinal cord along with neuronal sprouting may be the underlying mechanism of clinical improvement seen in this case. Patchy areas of sensory gain imply repair process, like remyelination, started at the neural fibers/tracts carrying sensations. Improvement in the bladder sensations was also recorded, as a result of myelination of sensory fibers after cellular therapy. The improvement in balance and lower limb control was due to strengthening of abdominals and back extensors. This may have led to improved gait along with enhanced functional status recorded as FIM even though change in ASIA grade was not recorded. This depicts that may be FIM is more sensitive to changes in functional status, whereas ASIA scale would be more appropriate to classify the patient. The patient had attained a plateau prior to this interventional approach, even with the ongoing rehabilitation. The improvements observed after the intervention may be attributed to the neurorestorative approach.

This case report is supported by multiple previous animal and human studies. In few of the animal studies done recently, it has been concluded that transplantation of stem cells into the lesioned spinal cord can lead to functional benefits [7, 8]. One of the recent studies was done, where human neuronal stem cells were transplanted into damaged spinal cord of a mouse. The cells generated new neurons and oligodendrocytes, leading to locomotor recovery [8]. Studies done in humans have also added to the evidence that autologous bone marrow MNCs help improve the functional outcomes in patients with chronic cervical and thoracolumbar spinal cord injuries [9, 10]. There is also a correlation observed between the implantation of stem cells, number of graft-derived oligodendrocytes, the amount of myelin, and the extent of functional recovery [11].

In this case study, autologous bone marrow mononuclear cells were the choice of cells over other types for the advantages they have over the others. Since they are autologous, use of these cells does not hold the risk of any graft versus host diseases or tumors. Another reason for use of these cells is that they have been found to have several types of bone marrow cells, including mesenchymal cells, hematopoetic cells, tissue specific progenitor cells, and stromal cells. These cells are known to produce large amounts of cytokines and trophic factors that promote angiogenesis, neuroprotection, and neuroregeneration. These cells were introduced intrathecally into the patients system. Intrathecal route was used for administration of cells, as it is a minimally invasive technique. It has also been found that administration intrathecally through the CSF may be a more targeted way of transmission than intravenous [12].

The unavailability of objective imaging to actually visualize the regeneration occurring at the level of injury before and after the neuroregenerative and neurorehabilitation treatment is one of the limitations. Further studies are needed to analyze more types of potent cells which can be incorporated and the best route for administering those cells. The possible dosage of these cells along with number of repetitions of these cell therapies with time interval between them also needs to be explored.

In conclusion, transplantation of autologous BMMNCs with neurorehabilitation seems to be an effective approach for improving functional outcomes in pediatric spinal cord injury. Though not a cure, cell therapy may have the potential for neurorestoration which is the underlying etiology of the disease. Since this was a single case report, it is preliminary evidence towards this neuroregenerative approach. It supports that cell therapy has the potential to improve the functional status, hence upgrading the quality of life of individuals with spinal cord injury. Further studies with larger and homogenous samples are needed to establish the results. Higher levels of studies such as randomized controlled trials are needed in this upcoming area of neuroregenerative research.
